# Integrated Analysis of MicroRNA and Target Genes in *Brachypodium distachyon* Infected by *Magnaporthe oryzae* by Small RNA and Degradome Sequencing

**DOI:** 10.3389/fpls.2021.742347

**Published:** 2021-10-01

**Authors:** Weiye Peng, Na Song, Wei Li, Mingxiong Yan, Chenting Huang, Yang Yang, Kangle Duan, Liangying Dai, Bing Wang

**Affiliations:** ^1^Hunan Provincial Key Laboratory for Biology and Control of Plant Diseases and Insect Pests, Hunan Agricultural University, Changsha, China; ^2^College of Plant Protection, Hunan Agricultural University, Changsha, China

**Keywords:** *Brachypodium distachyon*, *Magnaporthe oryzae*, miRNA, target, high-throughput sequencing

## Abstract

Rice blast caused by *Magnaporthe oryzae* is one of the most important diseases that seriously threaten rice production. *Brachypodium distachyon* is a grass species closely related to grain crops, such as rice, barley, and wheat, and has become a new model plant of Gramineae. In this study, 15 small RNA samples were sequenced to examine the dynamic changes in microRNA (miRNA) expression in *B. distachyon* infected by *M. oryzae* at 0, 24, and 48 h after inoculation. We identified 432 conserved miRNAs and 288 predicted candidate miRNAs in *B. distachyon*. Additionally, there were 7 and 19 differentially expressed miRNAs at 24 and 48 h post-inoculation, respectively. Furthermore, using degradome sequencing, we identified 2,126 genes as targets for 308 miRNAs; using quantitative real-time PCR (qRT-PCR), we validated five miRNA/target regulatory units involved in *B. distachyon–M. oryzae* interactions. Moreover, using co-transformation technology, we demonstrated that *BdNAC21* was negatively regulated by miR164c. This study provides a new approach for identifying resistance genes in *B. distachyon* by mining the miRNA regulatory network of host–pathogen interactions.

## Introduction

Plants are continuously exposed to a vast array of potential pathogens; however, only a small fraction of these pathogens can successfully infect and become established within the host plant species. This is because plants have evolved intricate defense mechanisms to protect themselves against invading pathogens (Wang et al., 2019). Successful initiation of plant defense responses against infectious pathogens requires complex and precise gene expression communication and reprogramming between plant and pathogen (Huang et al., 2019). Small RNA (sRNA)-mediated RNA interference (RNAi) is a well-conserved gene regulatory pathway that has emerged as an important regulator of reprogramming gene expression (Chang et al., 2012). Furthermore, sRNAs that participate in plant immunity responses can be classified as microRNAs (miRNAs) and small interfering RNAs (siRNAs) (Chen, 2012).

Mature miRNAs are short non-coding RNAs composed of 20–24 nucleotides (nts) derived from single-stranded RNA precursors with stem-loop structures (Bartel, 2004). In general, miRNAs negatively regulate target gene expression through mRNA cleavage, chromatin methyl modification, and/or translation inhibition (Baulcombe, 2004). Accurate and definite prediction of miRNA targets is essential for understanding miRNA responses. Degradome analysis facilitates the prediction and identification of miRNA targets based on the mechanism through which plant miRNAs have perfect or near-perfect complementarity with their targets (Garg et al., 2019). Recently, an integrated analysis of miRNAs and their targets has been reported. For example, using this analysis, nine miRNAs and their target genes have been shown to play crucial roles in peanut seed development (Ma et al., 2018).

Hence, a growing body of evidence indicates the importance of miRNAs in plant defense responses (Katiyar-Agarwal and Jin, 2010; Zhang et al., 2016). In *Arabidopsis*, miR393 is the first miRNA identified to contribute to resistance against virulent *Pseudomonas syringae* DC3000 (Navarro et al., 2006). In rice, miR398b-regulated Cu/Zn-Superoxide Dismutase 1 increased hydrogen peroxide accumulation to enhance resistance to *Magnaporthe oryzae*. Similarly, overexpression of *IPA1*—the target gene of miR156—enhanced resistance against bacterial blight caused by *Xanthomonas oryzae* pv. *oryzae* (*Xoo*) (Liu et al., 2019). Rice ragged stunt virus infection resulted in an increased accumulation of miR319 to reduce endogenous jasmonic acid (JA) levels (Zhang et al., 2016). Moreover, several miRNA families act as resistance regulators to mediate the silencing of nt-binding leucine-rich repeat type disease resistance genes (*R*-genes) in plants (Baldrich and San Segundo, 2016).

*Brachypodium distachyon* (L.) has been widely used as a model system for studying temperate grasses because of its relatively close evolutionary relationship with cereal grasses, such as wheat and rice (Draper et al., 2001). Additionally, *B. distachyon* possesses characteristics including a short life cycle, a small diploid genome, self-pollination, and low requirements for completing its growth successfully, which are desirable for a model system; hence, it has emerged as a promising resource for studies on plant–fungi interactions (Garvin et al., 2008; Parker et al., 2008). *M. oryzae* is the causative agent of rice blast, one of the most damaging fungal diseases of rice. The progression of *M. oryzae* disease in *B. distachyon* and rice is highly similar, including disease severity, size/shape of the lesions, duration from infection to appearance of symptoms and the expression of the pathogenesis-related genes (Routledge et al., 2004; Fitzgerald et al., 2015). An earlier report described that genome of *B. distachyon* contains *R*-genes that encode proteins with nucleotide-binding site and leucine-rich repeat domains. When these genes are transformed into rice, they confer resistance toward rice blast disease (Yang et al., 2013). In our earlier study, we showed that BdGATA transcription factors responded to invasion of *M. oryzae* (Peng et al., 2021). PAL-knockdown *B. distachyon* plants exhibited enhanced susceptibility to *M. oryzae* and the expression of genes associated with stress responses, including ethylene (ET) biosynthesis and signaling were significantly altered (Cass et al., 2015). In this study, we simultaneously performed miRNA sequencing and degradome sequencing to analyze the miRNAs and their targets involved in *B. distachyon*–*M. oryzae* interactions. Our results have provided useful data for further studies on the role of miRNAs in plant defense responses.

## Materials and Methods

### Plant Materials and Inoculation

*Brachypodium distachyon* genotype Bd21-3 was selected for this study. All plants, including control plants, were cultured in a growth chamber at 22°C under a 16/8 h photoperiod. Bd21-3 is compatible with *M. oryzae* race RO1-1. Approximately 5 weeks after sowing, the plants were inoculated with fresh *M. oryzae* spores (1 × 10^5^ spores mL^–1^) by spraying (Wang et al., 2020). Mock inoculation was performed in parallel with sterile water. Inoculated plants were placed in a moist chamber at 28°C in complete darkness for the first 24 h. Inoculated leaves and mock-treated control leaves were sampled at 0, 24, and 48 h post-inoculation (hpi) and snap-frozen in liquid nitrogen. Three independent biological replicates were used for each treatment.

### RNA Isolation and Small RNA Sequencing

Total RNA was extracted from *B. distachyon* leaves using TRIzol (Invitrogen, Carlsbad, CA, United States) according to the manufacturer’s instructions. RNA concentration was determined using a NanoDrop 1000 spectrophotometer (Thermo Fisher Scientific, MA, United States). Approximately 1 μg of total RNA was used to construct a sRNA library following the protocol of the mRNA-Seq sample preparation kit (Illumina, San Diego, CA, United States). Single-end sequencing (50 bp) was performed using an Illumina Hiseq2500 at the LC-BIO (Hangzhou, China) (Zhang et al., 2019).

### Identification of Known and Potential Novel MicroRNAs

Raw reads were processed with ACGT101-miR (LC Sciences, Houston, TX, United States) to remove adapters, junks, repeats, low-complexity sequences, and common non-coding RNA families (rRNA, tRNA, snRNA, and snoRNA). The remaining clean reads with lengths of 18--24 nts were blasted to miRBase 21.0^1^ to identify known miRNAs. Novel miRNA candidates were blasted to the genome of *B. distachyon*. The pre-miRNAs were mapped to specific species genomes to determine their genomic locations. The unmapped sequences were analyzed using BLAST against the *B. distachyon* genome. Then, hairpin RNA structures were predicted from the flank 120 nt sequences using RNAfold^2^ as previously described (Meyers et al., 2008). All raw sequencing data were deposited into the NCBI Short Read Archive under the BioProject: PRJNA751253 (Accession number: SRR15316624–SRR15316628).

### Degradome Library Construction and Target Identification

Approximately 20 μg of total RNA was used to prepare the degradome library (Zhai et al., 2020). Poly(A) + RNA was bound to mRNA capture beads as input RNA. Fifty adapters were ligated to RNAs containing 50-monophosphates, reverse transcribed, and PCR amplified. The purified cDNA library was used on Illumina’s Cluster Station and sequenced on an Illumina Hiseq 2500 at the LC-BIO. Raw sequencing reads were used to analyze potentially cleaved targets using the CleaveLand 3.0 pipeline.^3^ Differentially expressed miRNAs revealed by the normalized deep-sequencing read counts were analyzed by Student’s *t*-test. The threshold for significance was set at *p* < 0.05 and for high significance at *p* < 0.01 in each test.

### Gene Ontology and KEGG Analysis of Target Genes

The sequences of *B. distachyon* were obtained from Ensembl Plants^4^ genome-centric portal for plant species. We assigned genes to different categories based on the predicted functions of their *Arabidopsis* and rice homologs. BLAST2GO (Conesa et al., 2005) was then used to obtain Gene Ontology (GO) annotations at default settings of Fisher’s exact test (*p* < 0.05), false discovery rate (FDR) correction using the Hochberg method, and five minimum numbers of mapping entries against a species-specific pre-computed background reference. The KEGG13 pathway was analyzed using the ClueGO plug-in14 and Cytoscape software V2.8.215 to identify significant pathways of the differentially expressed genes (DEGs) (Bindea et al., 2009).

### Quantitative Real-Time PCR Analysis

Inoculated and control leaves were harvested at 0, 16, 48, and 120 hpi. First-strand cDNA was synthesized using a quantitative real-time PCR (qRT-PCR) system (Takara, Shiga, Japan) according to the manufacturer’s instructions. All qRT-PCR was performed on a CFX96 Real-Time System (Bio-Rad, Munich, Germany) using SYBR Green I (Thermo Fisher Scientific, Carlsbad, CA, United States). The total volume of PCR reaction was 25 μL. PCR conditions were as follows: 94°C for 5 min, 35 cycles (94°C for 30 s, 56°C for 30 s, and 72°C for 30 s) and 72°C for 5 min. UBC18 was used as an internal control for qRT-PCR analysis to normalize the data (Hong et al., 2008). The relative expression of *R*-genes was quantified using the comparative 2^–ΔΔ*CT*^ method (Voegele and Schmid, 2011). Primers for defense genes were designed using the DNAMAN 9 software (Supplementary Table 1). Three independent biological replicates were used for each treatment.

### Co-transformation of MicroRNAs and Target Genes

The region containing the cleavage site of BdNAC21 was cloned into the pBI121 vector containing the β-glucuronidase (GUS) reporter gene. The precursor of miR164c was integrated into the pBI121 vector to replace the GUS gene. Strain GV3101 of *Agrobacterium tumefaciens* was cultured in LB medium at 28°C to introduce the recombinant vectors into *Nicotiana benthamiana* leaves. Spectra of *A. tumefaciens* strains were normalized to an equivalent optical density of 0.8 at 600 nm (OD600) before injection. The strains mixed with pBI121-BdNAC21 and pBI121-miR164c were infiltrated into *N. benthamiana* leaves (Wang et al., 2017). Each treatment was infiltrated with 1 mL of the liquid containing the strains. Histochemical staining and quantitative GUS detection were performed was performed using 4-methylumbelliferyl-β-D-glucuronide (4-MUG) as the substrate (Jefferson et al., 1987).

β-Glucuronidase activities were measured using a Gemini XSP spectrophotometer plate reader (Molecular Devices, Sunnyvale, CA, United States) at wavelengths of 365 and 455 nm. Each assay was then performed in duplicate.

## Results

### Small RNA Sequencing of *Brachypodium distachyon*

**FIGURE 1:**
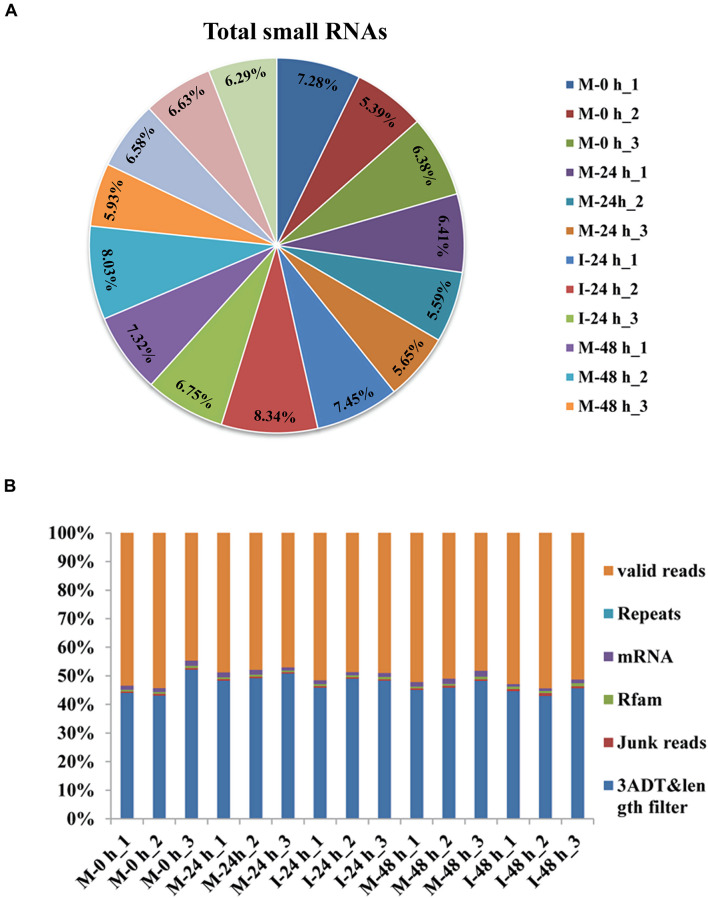
Profiling of small RNA sequencing in *B. distachyon* plants infected by *M. oryzae*. **(A)** Total clean reads-distribution and radioactivity (% of total) in libraries at each time point between each experimental group and the control group. **(B)** The classification distribution of the total small RNA in the five *B. distachyon* small RNA libraries.

**FIGURE 2:**
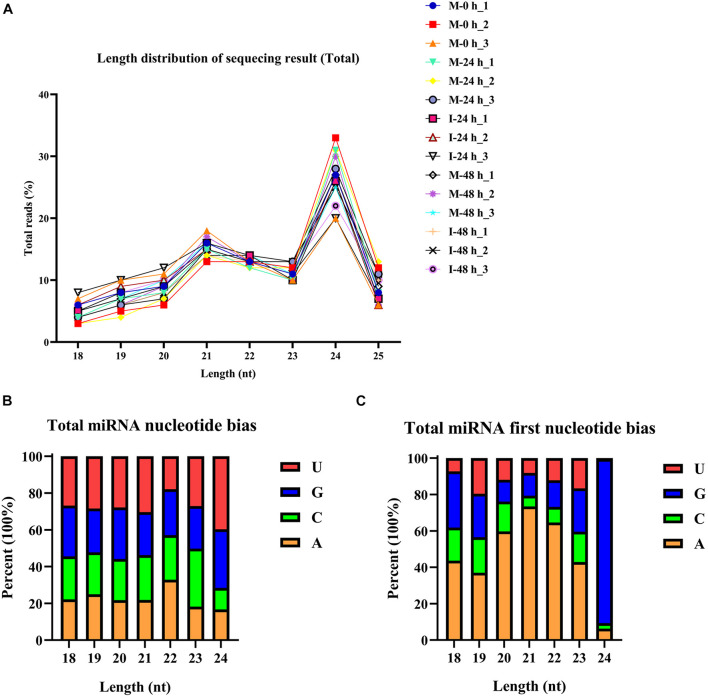
Total abundance of small RNA sequences in each size class and miRNA nucleotide bias analysis results. **(A)** Length distribution of sequencing results (total). **(B)** Results of total miRNA nucleotide bias. bdi is short for *Brachypodium distachyon*; gma is short for *Glycine max*; mdm is short for *Malus domestica*; osa is short for *Oryza sativa*; ptc is short for *Populus trichocarpa*; ath is short for *Arabidopsis thaliana*; mtr is short for *Medicago truncatula*; ata is short for *Aegilops tauschii*; gra is short for *Gossypium raimondii*; hvu is short for *Hordeum vulgare*; lja is short for *Lotus japonicus*; far is short for *Festuca arundinacea*. **(C)** Results of miRNA first nucleotide bias at each position.

The phenotype of *B. distachyon* infected with *M. oryzae* (isolate RO1-1) was identified. Large disease lesions were observed in susceptible *B. distachyon* plants (Bd21-3) 5 days after inoculation. The early stage in the infection of *B. distachyon* with *M. oryzae* is reportedly the key time point of the plant–fungus interaction. To validate whether sRNAs mediate the response of *B. distachyon* to *M. oryzae* infection, we examined the dynamic changes in miRNA expression in *B. distachyon* infected by *M. oryzae* at 0, 24, and 48 hpi using Illumina Hiseq2500. Fifteen sRNA libraries were constructed including M-0 h, M-24 h, I-24 h, M-48 h, I-48 h (mock and inoculation is abbreviated as M and I respectively; M-0 h: Mock 0 hpi; M-24 h: Mock 24 hpi; I-24 h: Inoculation 24 hpi; M-48 h: Mock 48 hpi; I-48 h: Inoculation 48 hpi; three biological replicates were performed per treatment). A total of approximately 13.6 million reads were obtained from each library (from 10,265,735 to 15,611,967 reads per library) (Figure 1A and Supplementary Table 2). After removing low-quality reads, unique reads (from 1,564,288 to 2,568,854 reads per library) were obtained (Figure 1B). The length distribution of the sRNAs ranging from 18 to 25 nt was further analyzed. sRNAs 24 nt in length were the most frequent, followed by sRNAs 21 nt in length (Figure 2A). We found that four types of nts had an average total miRNA nt bias (Figure 2B). Moreover, the first position in the 18–25 nt miRNAs was predominantly uracil (U), except for 24 nt miRNAs starting with adenine (A) (Figure 2C). Unique reads were annotated against miRBase and Rfam to eliminate rRNA, tRNA, and snoRNA (Supplementary Table 2).

### Identification of Novel and Known MicroRNAs

**FIGURE 3:**
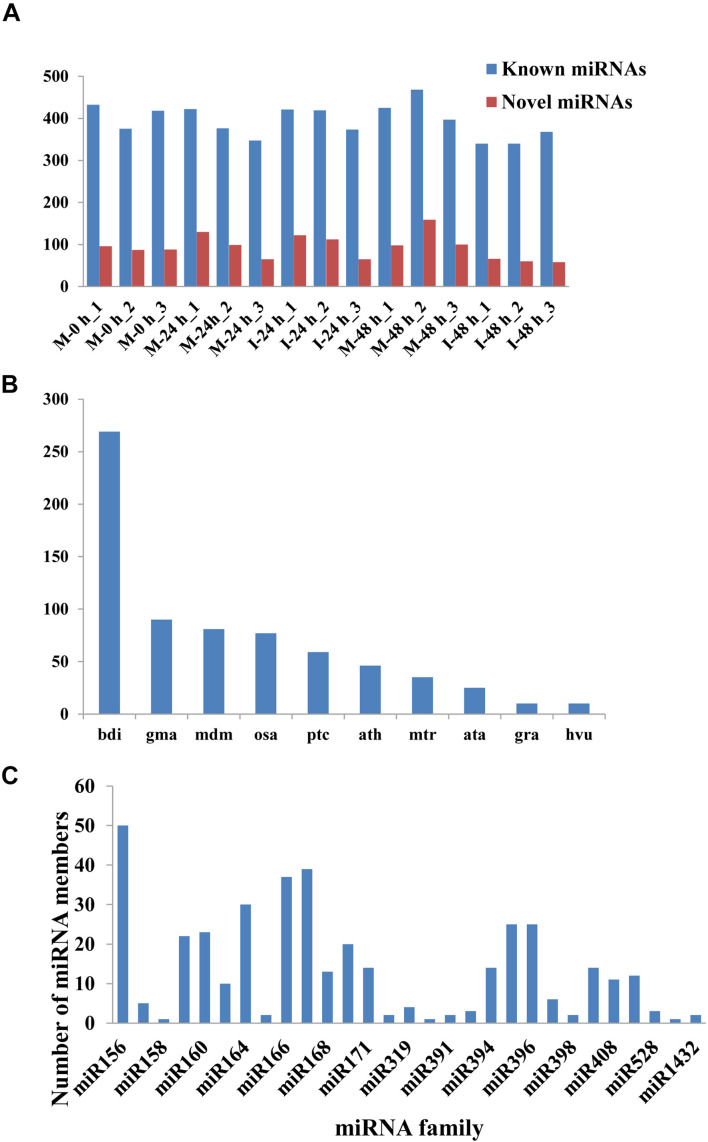
The statistics of miRNAs in the disparate sample. **(A)** Number of identified known and novel miRNAs. **(B)** Distribution of miRNA counts in different plant species. **(C)** Summary of the number of miRNA family members.

**FIGURE 4:**
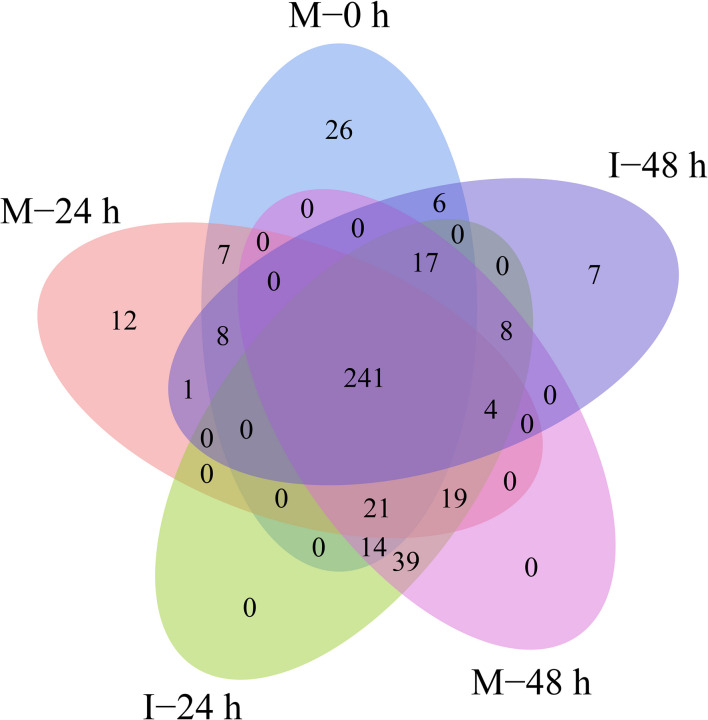
Venn-diagram analysis of the number and overlap of miRNAs in *B. distachyon* plants infected by *M. oryzae* at 0, 24, and 48 hpi.

A total of 432 known miRNAs were identified in the sRNA libraries by analyzing the unique clean reads that were aligned with miRBase (Figure 3A). These known miRNAs belonged to 77 miRNA families (Supplementary Table 3). We found that the miR156, miR164, miR166, and miR167 families contain more than 30 members. These known miRNAs were further compared to the data of 12 different plant species including *Arabidopsis thaliana* and *Oryza sativa* to explore their evolutionary characteristics (Figure 3B). As shown in Figure 3C, the 77 known miRNA families exhibited different numbers of homologous sequences in most of the comparisons. Fifteen known miRNAs were found to be conserved (Figure 3C). The remaining sequences that did not match any of the known miRNAs but mapped to the genome were then further analyzed based on whether they formed hairpins. A total of 288 predicted candidate miRNAs (PC miRNAs) were identified (Supplementary Table 4). Venn diagrams were created based on the comparison of five library miRNA sequences. The results showed that 26 and 12 miRNAs were only found in M-0 h and M-24 h groups, respectively. Moreover, seven miRNAs were unique to the I-48 h group (Figure 4).

### Identification of Differentially Expressed miRNAs in *Brachypodium distachyon* Plants Infected by *Magnaporthe oryzae*

**FIGURE 5:**
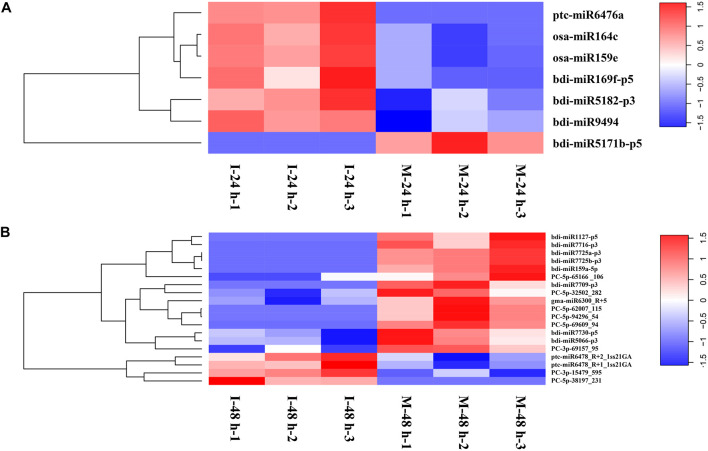
Heatmaps of differentially expressed miRNAs. **(A)** Heatmaps of differentially expressed miRNAs of *B. distachyon* leaves infected by *M. oryzae* at 24 hpi. **(B)** Heatmaps of differentially expressed miRNAs of *B. distachyon* leaves infected by *M. oryzae* at 48 hpi.

Differentially Expressed miRNAs (DEMs) examined at 24 hpi (M-24 h: Mock 24 hpi; I-24 h: Inoculation 24 hpi) and 48 hpi (M-48 h: Mock 48 hpi; I-48 h: Inoculation 48 hpi), yielded the absolute value of log2 fold change higher than 1.5 and a *p*-value below 0.05. We found seven DEMs at 24 hpi; six miRNAs were upregulated, whereas one miRNA was down-regulated (Figure 5A). Compared with M-48 h, we identified 19 DEMs in I-48 h, including four upregulated miRNAs and 15 downregulated miRNAs (Figure 5B and Supplementary Table 5). The expression of eight novel miRNAs changed at 48 hpi. Compared with M-0 h, we found 25 and 28 DEMs in *B. distachyon* infected by *M. oryzae* at 24 and 48 hpi, respectively (Supplementary Figure 1). Moreover, the number of DEMs between I-24 h and I-48 h was 25 (Supplementary Figure 1).

### Target Gene Identification and Function Analysis of MicroRNAs by Degradome Sequencing

To identify the target genes of miRNAs involved in the *B. distachyon*–*M. oryzae* interaction, a mixed degradome library was generated from the M-0 h, M-24 h, M-48 h, I-24 h, and I-48 h groups. Approximately 99.59% (24,200,004) sequenced raw reads of the degradome library were mappable when compared with genomic DNA; the number of unique mappable reads was 5,944,614. A total of 19,431,096 (79.96%) unique reads were mapped to the transcripts of *B. distachyon* protein-coding genes (Supplementary Table 6). Furthermore, strict conditions (category ≤2 and raw reads ≥10) were applied to screen the target genes to achieve accurate results for miRNAs and target genes in the degradome data library. A total of 2,126 genes were identified as targets for 308 miRNAs, including 258 known miRNAs and 49 novel miRNAs. The conserved miRNAs have multiple targets including transcription factors. Among the most conserved miRNAs and targets, we detected miR156a-R1-squamosa promoter-binding-like protein (SPL), miR164-NAC, miR396-growth-regulating factor (GRF), and miR444-MADS-box transcription factor (MADS). Novel miRNAs and targets were also detected, such as PC-3p-201628_13-THO2 (Supplementary Table 7).

**FIGURE 6:**
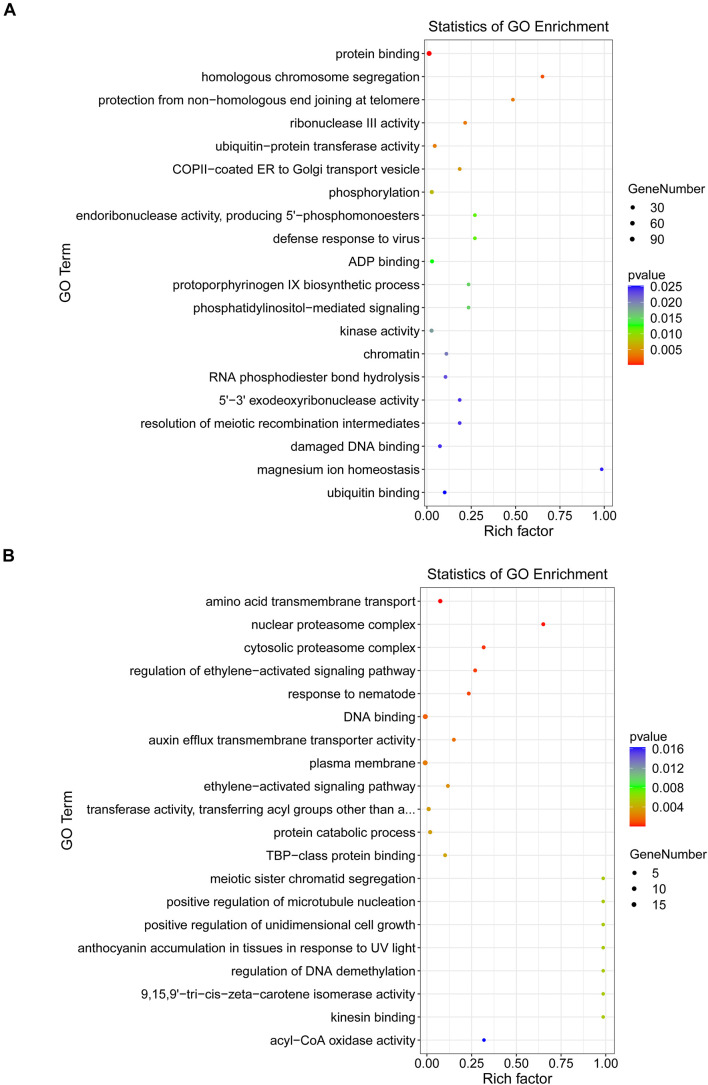
Gene Ontology (GO) term enrichments at 24 **(A)** and 48 **(B)** hpi.

To better understand the regulatory roles of DEMs and their targets in the interaction between *B. distachyon* and *M. oryzae*, GO enrichment annotations were performed at 24 and 48 hpi. GO enrichment analysis revealed that there were significantly enriched processes involved in plant defense responses, such as JA and ethylene-dependent systemic resistance. Furthermore, acyl-CoA oxidase and kinesin-binding activities were among the significantly enriched processes at 24 hpi (*p* < 0.05) (Figure 6A). At 48 hpi, ubiquitin-protein transferase activity and kinase activity were among the significantly enriched processes (Figure 6B). Moreover, to elucidate the exact biological process by which DEGs might participate in plant–fungus interactions, we performed a KEGG enrichment analysis. Our results revealed that “peroxisome” and “ubiquitin mediated proteolysis” were significantly enriched pathways involved in plant defense responses at 24 and 48 hpi, respectively (Supplementary Figure 2 and Supplementary Table 8).

### Quantitative Real-Time PCR Validation

**FIGURE 7:**
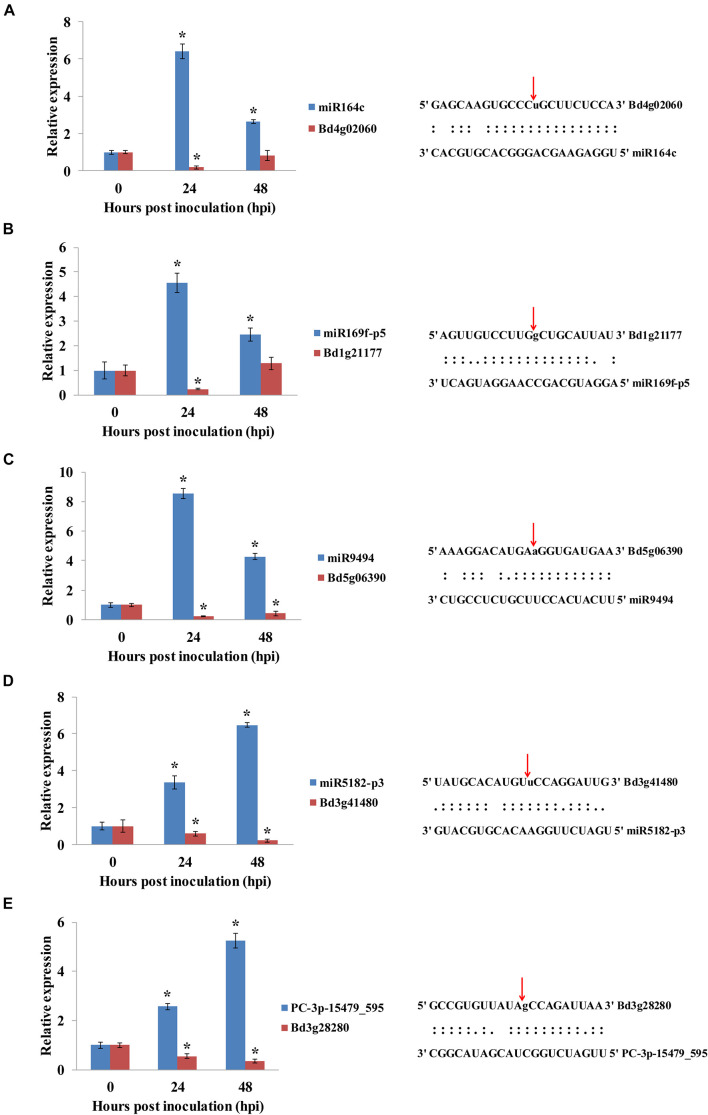
**(A–E)** qRT-PCR analysis of the expression of miRNAs and their corresponding targets during *B. distachyon–M*. *oryzae* interaction. The expression of mRNAs and their corresponding targets were analyzed by qRT-PCR. Asterisk (*) represents statistically significant difference (*P* < 0.05). Blue columns indicate miRNAs, and red columns indicate their corresponding targets. Expression values were normalized by UBC18 expression.

To validate the miRNA/target regulatory unit, the changes in expression of miRNAs and mRNAs during the *B. distachyon*–*M. oryzae* interaction were analyzed using qRT-PCR. Five miRNAs, including one novel miRNA and four known miRNAs (Figure 7) were randomly selected to show cleavage events *via* degradome sequencing. DEMs and their corresponding targets were selected for qRT-PCR analysis. Consistent with the analysis of sequencing data for miRNAs, five target genes were negatively regulated by the corresponding miRNAs. Moreover, miR164c/*Bd4g02060* (*BdNAC21*), miR169-p5/*Bd1g21177* (*BdMED12*), and miR9494/*Bd5g06390* (*BdFKBP65*) were mainly regulated at 24 hpi. However, miR5182-p3/*Bd3g41480* (*BdPDH2*) and PC-3p-15479/*Bd3g28280* (*BdPRP19*) were more significantly regulated at 48 hpi than at 24 hpi.

### Co-transformation Analysis

**FIGURE 8:**
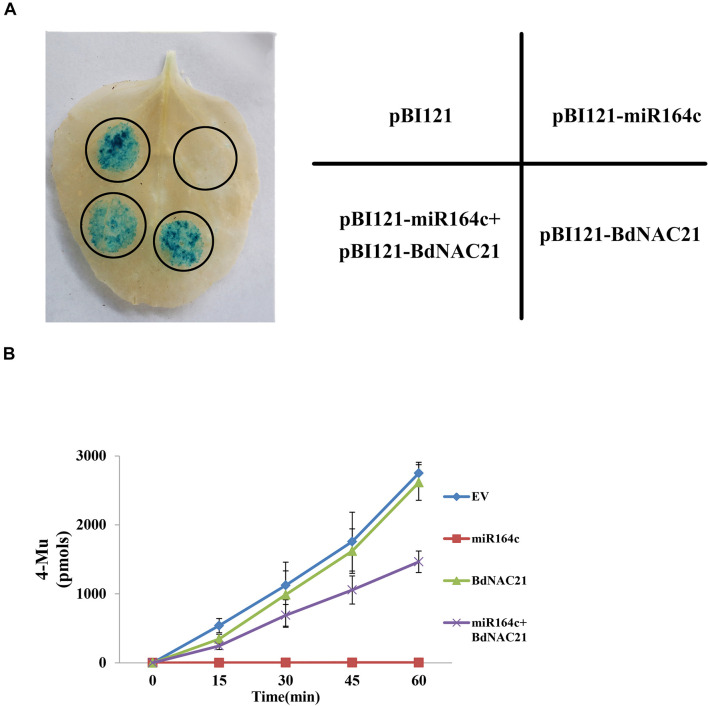
Co-transformation of BdNAC21 and miR164c in *N. benthamiana* leaves. Recombinant vectors were carried into *N. benthamiana* leaves by Agrobacterium strain GV3101. **(A)** Histochemical staining of GUS activity. **(B)** Quantitative analysis of GUS fluorescence.

To confirm the suppression of *BdNAC21* by miR164c, we performed transient co-transformation of *N. benthamiana* (Figure 8A). The recombinant vector pBI121 contained GUS as a reporter gene. Leaves infiltrated with pBI121-*BdNAC21* showed the GUS staining. In leaves infiltrated with mixture of pBI121-*BdNAC21* and pBI121-miR164c, GUS staining was reduced. The amount of GUS in each leaf was also assessed to further validate results of the histochemical observations (Figure 8B). We found that the fluorescence from the empty vector and pBI121-*BdNAC21* was enhanced on extending the reaction time for the inoculated leaf samples. No fluorescence was observed in leaves injected with pBI121-miR164c. However, the fluorescence of the leaf injected with the mixture strain (pBI121-*BdNAC21* and pBI121-miR164c) exhibited a slow increase. These results further confirm that *BdNAC21* is the gene targeted by miR164c.

## Discussion

*Magnaporthe oryzae* is the causal agent of rice blast. This fungus can also infect *B. distachyon* and the disease progression is similar to that observed in rice (Fitzgerald et al., 2015). We have previously found that multiple genes are involved in the interaction between *M. oryzae* and *B. distachyon*. In general, miRNAs regulate target genes involved in multiple biological processes in plants. For example, 16 BdGATA genes were predicted targets of 13 miRNAs (Peng et al., 2021). We inferred that miRNAs might regulate their targets during the *M. oryzae*–*B. distachyon* interaction.

To confirm whether miRNAs mediate the *B. distachyon* response to *M. oryzae*, we constructed 15 sRNA libraries. The 18–25 nt sRNAs were analyzed. We found that 24 nt miRNAs were the most frequent form, followed by 21 nt sRNAs. These patterns of length distribution have also been identified in rice and barley (Hackenberg et al., 2015; Jia et al., 2020). Moreover, the first position in 18–25 nt miRNAs was predominantly U. Usually, miRNAs are loaded into the RNA-induced silencing complex having a high affinity to U, which is at the first position in these miRNAs (Mi et al., 2008).

In this study, a total of 432 known miRNAs belonging to 77 miRNA families were identified. Evolutionary analysis revealed that most of these known miRNAs were enriched in 15 conserved miRNA families, including miR156, miR164, miR166, and miR167. Transgenic lines expressing a target mimic of miR156fhl-3p displayed enhanced resistance to *M. oryzae* by induction of pathogenesis-related gene expression without yield penalty (Zhang et al., 2020). In addition, miR164, miR166, and miR168 were all reported to be involved in the rice immune response against *M. oryzae* infection (Salvador-Guirao et al., 2018; Wang et al., 2021). A total of 288 predicted PC miRNAs were identified. Furthermore, we found that 26, 12, and 7 miRNAs were only found in M-0 h, M-24 h, and I-48 h, possibly indicating that miRNAs could be targeted functionally at specific time points during infection. Recently, the use of deep sequencing has allowed additional non-conserved miRNAs to be identified (Feng et al., 2016). These predicted PC miRNAs were identified at relatively low abundance and expressed in specialized treatments (Fahlgren et al., 2007).

A set of miRNAs was found to be involved in plant immunity (Li et al., 2014). We identified 7 and 19 DEMs at 24 and 48 hpi, respectively. Furthermore, we identified the target genes of miRNAs by degradome sequencing, such as miR396-*BdGRF* and miR444-*BdMADS*. Osa-miR396 regulates *OsGRFs* involved in rice resistance to *M. oryzae* (Chandran et al., 2019). Overexpression of miR444-resistant *OsMADS57* rice enhanced resistance against rice stripe virus infection (Wang et al., 2016). Moreover, we observed that *BdNAC21* was negatively regulated by miR164c during the *M. oryzae*–*B. distachyon* interaction. In rice, miR164a can impair plant resistance to *M. oryzae* infection by inhibiting the expression of *OsNAC60* (Wang et al., 2018). Similarly, overexpression of osa-miR164a plants weakened the resistance to the *Xoo* strain owing to the reduced accumulation of *OsNAC60* (Jia et al., 2020). These results indicate that there may be conserved miRNA regulatory modules acting in plant resistance responses to fungi.

We performed GO enrichment and KEGG analyses of the target genes of DEMs. Some pathways are involved in plant defenses, such as ubiquitin-protein transferase activity and kinase activity. Interestingly, the target genes of DEMs fell within the JA and ethylene-dependent systemic resistance at 24 hpi, which can induce systemic resistance in plants (Niu et al., 2011). In the ptc-miR472a-overexpressing poplar plants, the JA/ethylene marker gene *PtrERF1* displayed very high expression levels and promoted the JA/ET signal to rapidly respond to the necrotrophic fungus *Cytospora chrysosperma* (Su et al., 2018). Moreover, one of the most enriched KEGG pathways was “peroxisome.” Coordination of the biosynthesis, degradation, biochemical activity, and import of peroxisomal proteins allows for highly dynamic responses of peroxisomal metabolism to provide resistance to abiotic and biotic stress (Hu et al., 2012). Using iTRAQ proteomics analysis for discovery, the differentially expressed proteins involved in peroxisome biosynthesis were significantly different between the durable resistant rice variety and the susceptible rice variety (Ma et al., 2020). The KEGG pathway analysis also demonstrated that the processes of biosynthesis, metabolism, nucleotide excision or base excision repair were enriched at 48 hpi, suggesting accelerated energy compensation and maintenance/repair processes at the two infection stages. These results suggest that miRNAs regulate complex pathways involved in plant defense responses.

Here, we identified 432 conserved miRNAs and 288 PC miRNAs in *B. distachyon*. There were 7 and 19 DEMs at 24 and 48 hpi, respectively. We identified 2,126 genes as targets for 308 miRNAs. The expression levels of five miRNAs and target units were validated by qRT-PCR. Using co-transformation technology, we have clearly demonstrated that BdNAC21 is negatively regulated by miR164c. These results provide a comprehensive foundation for unraveling complex miRNA-mediated regulatory networks at play during the interaction between *M. oryzae* and *B. distachyon*.

## Data Availability Statement

The datasets presented in this study can be found in online repositories. The names of the repository/repositories and accession number(s) can be found below: NCBI SRA BioProject, accession no: PRJNA751253.

## Author Contributions

WP were responsible for study initiation and performed the experiments. NS analyzed the data and wrote the first draft of the manuscript. MY, YY, CH, KD, and WL contributed to the literature search, collection and assembly of data, and contributed the reagents. LD and BW designed the experiments, supervised the project, and grammar correction. All authors contributed to the article and approved the submitted version.

## Conflict of Interest

The authors declare that the research was conducted in the absence of any commercial or financial relationships that could be construed as a potential conflict of interest.

## Publisher’s Note

All claims expressed in this article are solely those of the authors and do not necessarily represent those of their affiliated organizations, or those of the publisher, the editors and the reviewers. Any product that may be evaluated in this article, or claim that may be made by its manufacturer, is not guaranteed or endorsed by the publisher.

## Footnotes

1
ftp://mirbase.org/pub/mirbase/CURRENT/

2
http://rna.tbi.univie.ac.at/

3
http://sites.psu.edu/axtell

4
http://plants.ensembl.org/Brachypodium_distachyon/Info/Index
