# Supplementary figures and images for: Integrated Analysis of MicroRNA and Target Genes in Brachypodium distachyon Infected by Magnaporthe oryzae by Small RNA and Degradome Sequencing
Source: Front Plant Sci. 2021 Oct 1;12:742347. doi: 10.3389/fpls.2021.742347 (PMC8517397; doi:10.3389/fpls.2021.742347)

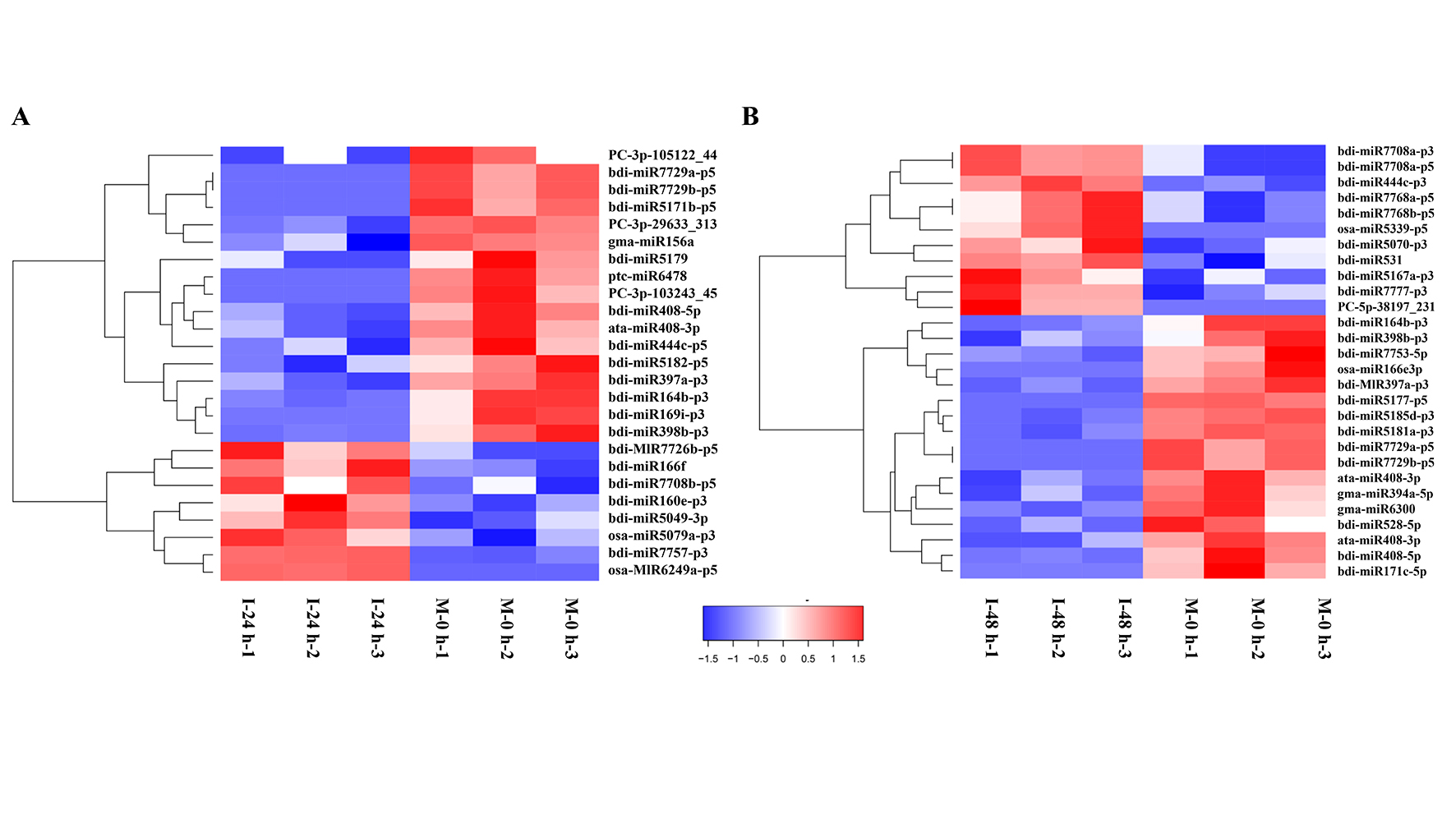

Supplement: Supplementary file 1 [file Image_1.JPEG]

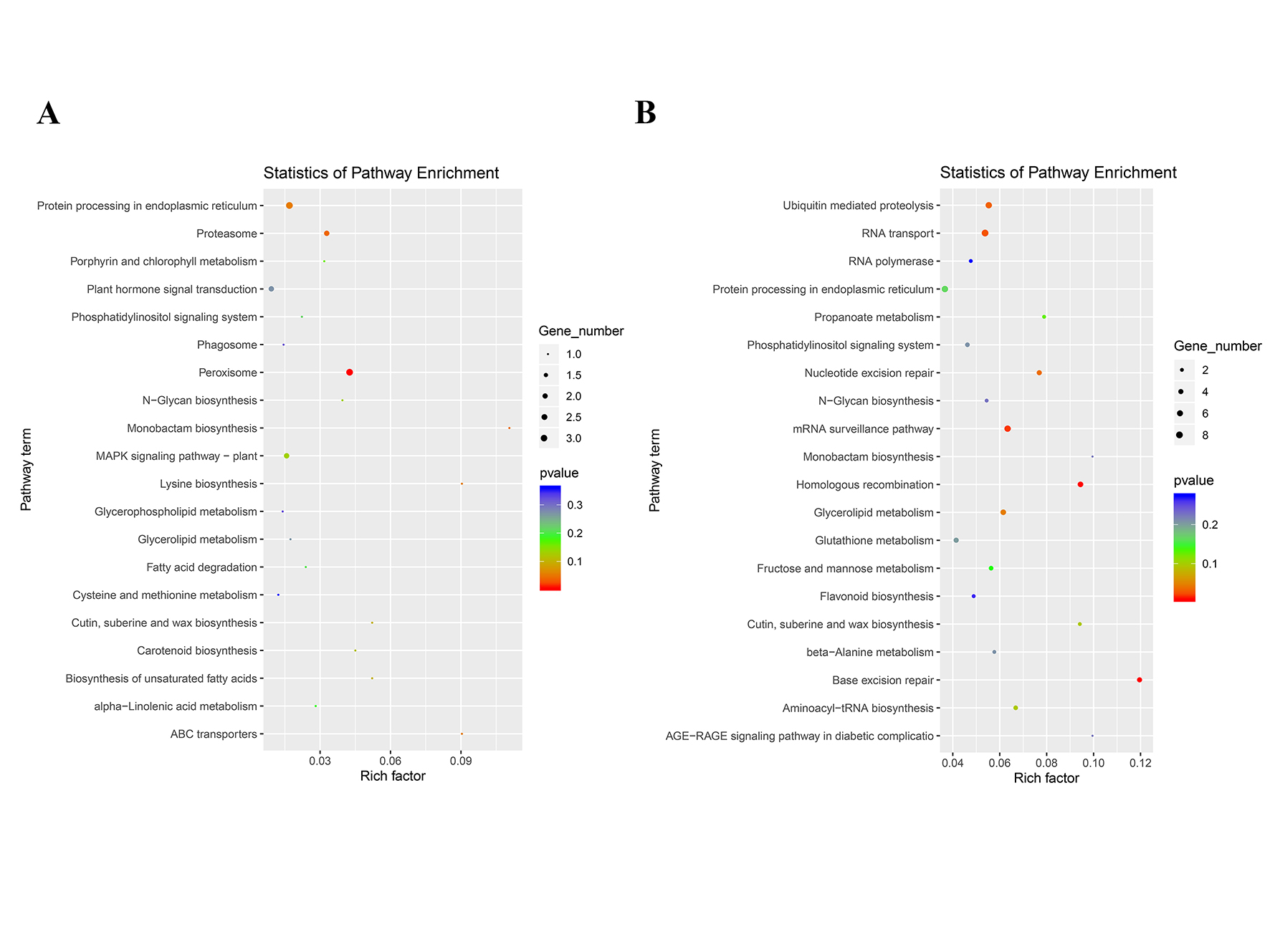

Supplement: Supplementary file 2 [file Image_2.JPEG]
